# Estimates of Sequences with Ultralong and Short CDR3s in the Bovine IgM B Cell Receptor Repertoire Using the Long-read Oxford Nanopore MinION Platform

**DOI:** 10.4049/immunohorizons.2400050

**Published:** 2024-09-09

**Authors:** Tess E. Altvater-Hughes, Harold P. Hodgins, Douglas C. Hodgins, Natasha B. Gallo, Gabhan I. Chalmers, Nicole D. Ricker, Bonnie A. Mallard

**Affiliations:** * Department of Pathobiology, Ontario Veterinary College, University of Guelph, Guelph, Ontario, Canada; † Department of Biology, University of Waterloo, Waterloo, Ontario, Canada

## Abstract

Cattle produce Abs with an H chain ultralong CDR3 (40–70 aa). These Abs have been shown to have features such as broad neutralization of viruses and are investigated as human therapeutics. A common issue in sequencing the bovine BCR repertoire is the sequence length required to capture variable (V) and isotype gene information. This study aimed to assess the use of Oxford Nanopore Technologies’ MinION platform to perform IgM BCR repertoire sequencing to assess variation in the percentage of ultralong CDR3s among dairy cattle. Blood was collected from nine Holstein heifers. B cells were isolated using magnetic bead–based separation, RNA was extracted, and IgM^+^ transcripts were amplified using PCR and sequenced using a MinION R10.4 flow cell. The distribution of CDR3 lengths was trimodal, and the percentage of ultralong CDR3s ranged among animals from 2.32 to 20.13% in DNA sequences and 1.56% to 17.02% in productive protein sequences. V segment usage varied significantly among heifers. Segment *IGHV1-7*, associated with ultralong CDR3s, was used in 5.8–24.2% of sequences; usage was positively correlated with ultralong CDR3 production (*r* = 0.99, *p* < 0.01). To our knowledge, this is the first study to sequence the bovine BCR repertoire using Oxford Nanopore Technologies and demonstrates the potential for cost-efficient long-read repertoire sequencing in cattle without assembly. Findings from this study support literature describing the distribution of length and percentage of ultralong CDR3s. Future studies will investigate changes in the bovine BCR repertoire associated with age, antigenic exposure, and genetics.

## Introduction

Unusual subsets of Abs have been identified in multiple species, from Ig new Ag receptors in cartilaginous fish (1), single-domain Abs in camelid species (2), to Abs with an H chain (HC) ultralong CDR3 in cattle (3, 4). Abs in cattle have a conventional Ig structure with two HCs and two L chains (LCs) containing constant and variable domains. The variable domain comprises CDR1, CDR2, and CDR3 interspersed among framework regions (FWRs) 1–4.

Whereas typical human CDR3s range in length from 3 to 20 aa (5), the range in cattle is much larger (4). The distribution of CDR3 length in cattle has been commonly described as bimodal (conventional CDR3 [5–38 aa] and ultralong CDR3 [≥40–70 aa]) (6). However, it has also been reported as trimodal with three distinct peaks, including short CDR3s (≤10 aa) (7, 8), medium CDR3s, and ultralong CDR3s (≥40 aa) (4, 7, 8).

The ultralong CDR3s were first reported in 1997 by Berens et al. (3), but were further described and characterized by Lopez et al. (9) and Saini et al. (4). The ultralong CDR3 forms an atypical structure with an ascending β-ribbon stalk protruding from the Ab, followed by a folded knob domain and a descending stalk structure (10, 11). The knob domain contains multiple somatic hypermutation (SHM) hotspots (RGYW/WRCY) located throughout the CDR3 that are one nucleotide away from coding for a cysteine (6, 12). Cysteines tend to appear in even numbers and form disulfide bonds throughout the knob domain, increasing structural diversity (4, 11, 13). A CDR3 of this magnitude is unique to *Bos* and *Bison* genera and has not been reported in the Ab repertoires of other genera (14). Previous estimates of the percentages of ultralong CDR3s vary in the literature from 0.036 to 13% (4, 6, 13, 15, 16). The wide reported range in percentages of ultralong CDR3s may be due to different sample types, populations, antigenic pressure, individual genetics, and sequencing platforms.

The Ag binding region of BCR and Abs is composed of variable (V), diversity (D), and joining (J) genes. In cattle, there are 12 functional V genes, 16 functional D genes, and 4 functional J genes (4, 9, 12, 17). Compared to humans, the number of bovine functional VDJ genes is limited (18). It is noteworthy that cattle preferentially use specific VDJ genes to produce ultralong CDR3s (4, 12, 19). Typically, *IGHV1-7*, which encodes an 8-bp repeat, comprises the ascending β-strand of the stalk of ultralong CDR3s. The knob domain is attributed to the usage of the *IGHD8-2* gene (19), which encodes 148 bp with ∼19 SHM hotspots (6). The J segment *IGHJ2-4* is used in most Ab sequences (∼95% of bovine Ab sequences, including ultralong CDR3s) (7) and contributes to the ultralong CDR3 descending strand found in the stalk. It is theorized that ultralong CDR3s compensate in part for the lack of VDJ diversity through hypermutated CDR3s and structural diversity, which may be crucial to control the broad spectrum of microbes present in the rumen and to confront viral pathogens (12).

Bovine Abs with ultralong CDR3s have been reported to mediate broad neutralization of viruses, including human-specific viruses. Sok et al. (20) have reported that vaccination of cattle with recombinant HIV SOSIP gp140 trimers induced rapid production of virus-neutralizing Abs against HIV with the ability to bind hidden and concave epitopes. The use of engineered bovine ultralong CDR3s has been recently investigated against human SARS-CoV-2 (21). Humans do not typically produce Abs with CDR3s of this length nor Abs with the unique structure and functional characteristics of bovine Abs with ultralong CDR3s (11). The bovine ultralong CDR3s are, therefore, of intense interest for human therapeutic uses and Ab engineering (22, 23). Understanding the fundamental aspects of the expression of ultralong CDR3 Abs in cattle such as effects of age, health status, and routine vaccination is also important for applications and management decisions within the cattle industry. The functionality of short HC-CDR3s in cattle is unknown but deserves more attention. In humans and mice, studies of certain clonotypes with short HC- and LC-CDR3s have shown neutralization against viral epitopes using mainly CDR1 and CDR2, with little interaction with the CDR3 (24, 25).

A crucial tool in studying bovine BCR repertoires is sequencing platforms. Sequencing platforms such as Pacific Bioscience (PacBio) and Oxford Nanopore Technologies (ONT) offer long-read sequencing (LRS), whereas companies such as Illumina offer short-read sequencing (SRS). Based on the literature, the length of the bovine ultralong CDR3 itself can range from 120 to 210 bp, not including V gene sequences upstream from CDR3 or isotype-specific sequences downstream from CDR3. Amplicons of interest for this study ranged from 700 to 1200 bp. Therefore, current SRS technology has limitations, especially for assembling ultralong CDR3 sequences while maintaining V gene and isotype sequence associations spanning a potentially heavily mutated CDR3. The LRS platforms provide short to long reads (>400 kb), avoiding the need to assemble sequences (26). However, LRS is conventionally considered to be lower throughput, more expensive, and more error-prone than SRS platforms (26). Recently, the new V14 chemistry and R10.4 technologies from ONT are affordable and have a reported raw read accuracy of >99%, making ONT a potentially valuable and cost-efficient method of sequencing full-length CDR3 transcripts in cattle (26, 27). Due to the variability in CDR3 length paired with the interest in the variability of length among animals, LRS provided a reliable approach.

The primary objective of this study was to assess the use and feasibility of ONT for sequencing the bovine BCR repertoire and providing estimates of the length of CDR3s in Canadian Holsteins. In addition, the current study provides estimates of the percentage of blood B cells expressing ultralong CDR3s and evaluates V, D, and J gene usage.

## Materials and Methods

### Animals and sampling

The Animal Care Committee at the University of Guelph approved all animal use in this study (Animal Use Protocol no. 4449). Animals were housed at the Ontario Dairy Research Centre near Elora, Ontario. Forty milliliters of whole blood was collected in K_2_EDTA-coated blood tubes (Becton Dickinson, Franklin Lakes, NJ, catalog no. 02-657-32) from nine purebred Holstein-Friesian heifers. All heifers were housed in the same barn with a median age of 12.4 (±4.2) mo. Three heifers were bred and pregnant at sampling; the remaining six heifers were not yet bred. All pregnant heifers were <5.5 mo pregnant.

### Isolation of blood mononuclear cells

All centrifugation steps were performed at room temperature (RT). A summary flowchart of laboratory methods can be found in Supplemental Fig. 1. Whole blood was centrifuged for 15 min at 1200 × *g*, and the buffy coat was collected and suspended in 15 ml of PBS (Wisent, Saint-Jean-Baptiste, QC, Canada, catalog no. 311-425-CL). Using an underlay of Histopaque-1077 (Sigma-Aldrich, St. Louis, MO, catalog no. 10771) for density centrifugation, cells were centrifuged at 1200 × *g* for 15 min. The mononuclear cell portion was collected and suspended in 50 ml of PBS and centrifuged at 200 × *g* for 20 min. The pellet was then washed twice using PBS and centrifuged at 400 × *g* for 5 min. Any remaining RBCs were lysed using sterile water, and the PBMC portion was suspended in PBS and centrifuged at 400 × *g* for 5 min. The PBMCs were counted, and cell viability was assessed using a Corning CytoSMART cell counter (Corning Life Sciences, Corning, NY).

### MACS for positive B cells

Sorting of IgM^+^ cells was completed to enrich the B cell population for RNA extraction. Enriching for B cells would increase the yield of relevant sequences and improve the efficiency of PCR, which was cost-efficient compared with single-cell sorting.

Mononuclear cells (1 × 10^7^) were incubated at RT for 15 min with 250 µl of a 1:50 dilution of mouse IgG1 anti-bovine IgM (Sigma-Aldrich, catalog no. I6137, clone BM-23, 4 µg/ml) in PBS. Cells were washed twice with MACS buffer (PBS with 0.5% BSA and 2 mM EDTA) and centrifuged for 5 min at 400 × *g*. Next, cells were incubated in the dark at 4°C for 15 min with rat anti-mouse IgG1 microbeads (20 μl of microbeads and 80 μl of MACS buffer for 10^7^ PBMCs (Miltenyi Biotec, Bergisch Gladbach, Germany, catalog no. 130-047-102). Cells were then washed twice and sorted using a positive selection method. Cells were added to a MACS mini-column (Miltenyi Biotec, catalog no. 130-042-201) per the manufacturer’s instructions. The positively (IgM^+^) sorted cells were collected and centrifuged for 5 min at 400 × *g* and the supernatant was removed. Samples were then resuspended in 1 ml of TRIzol (Invitrogen, Waltham, MA, catalog no. 15596026) and then stored at −80°C.

### cDNA and PCR

Samples were thawed on ice, and 200 μl of chloroform was added and samples were mixed. Samples were centrifuged at 16,089 × *g* for 15 min at 4°C, and then the aqueous phase containing RNA was collected. Next, 500 μl of isopropanol (≥99.5%) was added, and the samples were vortexed and then incubated at −20°C overnight. Samples were centrifuged at 16,089 × *g* for 10 min at 4°C, the supernatant was removed, and the RNA pellet was washed twice with 75% ethanol. The supernatant was removed, and the RNA was resuspended in 12 μl of nuclease-free water. Any genomic DNA contamination was removed using the Turbo DNA-free kit (Invitrogen, catalog no. AM1907). An Agilent 4150 TapeStation system was used to ensure an RNA integrity number ≥8 during optimization. The quality and quantity were checked using a DeNovix spectrophotometer/fluorometer (DS-11) to ensure a 260/280 nm absorbance ratio ≥1.8. Using 5000 ng of RNA and the SuperScript III first-strand synthesis system (Invitrogen, catalog no. 18080051), cDNA was produced. In brief, the RNA, dNTP, diethyl pyrocarbonate (DEPC)–treated water, and oligo(dT)_20_ were mixed and incubated at 65°C for 5 min, then placed on ice. The RT buffer, MgCl_2_, DTT, RNase out, and SuperScript III reverse transcriptase were added and incubated for 50 min at 50°C, and the reaction was terminated at 85°C for 5 min and placed on ice. RNase H was added, and the tubes were incubated for 20 min at 37°C. Samples were stored at −20°C until they were used for downstream PCR applications.

The PCR reaction was prepared using the high-fidelity PCR master kit (Roche, Indianapolis, IN, catalog no. 12140314001) and adapted from the manufacturer’s protocol. First, 2 μl of cDNA was placed in a Roche 96-well plate (catalog no. 04729692001), after which 23 μl of mixed forward primer (300 nM), reverse primer (300 nM), and diethyl pyrocarbonate–treated water was added. Next, 25 μl of the high-fidelity master mix was added to each reaction. The forward primer (5′-AGATGAACCCACTGTGGACC-3′) was used to amplify all *IGHV* genes unbiasedly, adapted from a forward primer used by Saini et al. (18). The reverse primer (5′-TGTTTGGGGCTGAAGTCC-3′) was used to amplify IgM HC regions, adapted from Ma et al. (17). When analyzing the reverse primer from Saini et al. (18), there was evidence that the primer was not specific for IgM because the reverse primer sequence was identified in published IgD sequences (accession no. AF515672). In addition, the Saini et al. (18) primer was only 5 nt different from the published IgG sequence. The initial denaturation step was completed at 94°C for 2 min for 1 cycle. For the amplification cycles, the denaturation step occurred at 94°C for 15 s, annealing at 62°C for 30 s, and the elongation step at 72°C at 45 s for 10 cycles and then repeated for 10 more cycles while adding 5 s for each successive cycle of the elongation step. The final elongation step was completed at 72°C for 7 min.

During the optimization, PCR products were evaluated by gel electrophoresis to ensure no genomic DNA contamination and that PCR products were within the estimated base pair length. The PCR amplicons were then cleaned and purified using the PureLink PCR purification kit (Invitrogen, catalog no. K310001). Quantity and quality were rechecked to ensure a concentration of ≥20 ng/μl and a 260/280 nm absorbance ratio ≥1.8, and samples were stored at −20°C until sequencing was completed.

### MinION sequencing

End preparation was completed by preparing 1 μg of the amplicon reaction with the NEBNext Ultra II end repair/dA-tailing module (New England Biolabs, Ipswich, MA, catalog no. E7546S). Samples were washed and eluted in nuclease-free water. Samples were then barcoded with nine separate barcodes and prepared using the Native Barcoding Kit 24 v14 (Oxford Nanopore Technologies, Oxford, UK, catalog no. SQK-NBD114.24) and the Blunt/TA ligase master mix (New England Biolabs, catalog no. M0367S). Samples were washed and eluted in nuclease-free water. Barcoded samples were then pooled, and adapters were added using the NEBNext quick ligation module (New England Biolabs, catalog no. E6056S) and the ONT Ligation Sequencing Kit v14 (SQK-LSK114). The pooled barcoded sample was washed and eluted in nuclease-free water. Then, 75 µl of the pooled nine barcoded sample was added to the MinION on the same R10.4 flow cell (Oxford Nanopore Technologies, catalog no. R10.4.1) and allowed to run for ∼72 h.

### Analysis

#### Cleaning

Once FAST5 files were generated and sequencing was concluded, basecalling and barcode sorting/trimming and adapter removal were completed using ONT Guppy basecaller software (v6.4.6+ae70e8f) (–flowcell FLO-MIN114,–kit SQK-LSK114, –detect_barcodes, –enable_trim_barcodes). The fastq files containing sequence ID, phred score, nucleotide sequence, and comments were retrieved for each barcoded sample. The quality of the sequences was checked using the FASTQC software (v0.11.9). The sequences were quality filtered using the default parameters of fastp (v0.23.1) for quality (Q ≥ 15). Sequences were also filtered by length (–length_required 700 –length_limit 1200). Raw fastq files can be found in the NCBI Sequence Read Archive under BioSample accession no. PRJNA1044413.

The minimum sequence length (700 nt) captures sequences from the forward primer to a well-defined 21-nt IgM motif from Walther et al. (28) (Table I), corresponding to a very short to nonexistent CDR3. The maximum size (1200 nt) captures sequences with the forward primer, the 21-nt IgM motif from Walther et al. (28), and may include the reverse primer with an ultralong CDR3 (defined as up to 220 nt between the pre- and post-CDR3 motifs; see motif definitions below).

**Table I.** Descriptive filtering steps of the refined stepwise literature-based filtering method

Step	Search	Filter (5′→3′)	Parameters	Description	Reasoning
1	Quality filtering	—	—	Sequences were filtered for quality using fastp (v0.23.1) for a mean score of >Q15 Sequences <700 nt in length were removed Sequences >1200 nt in length were removed	Sequences were filtered by length to capture reads that were 700–1200 nt. The minimum sequence length captures sequences from the forward primer to a well-defined 21-nt IgM motif from Walther et al. (28), corresponding to a very short to nonexistent CDR3. The maximum size captures sequences with the forward primer, the 21-nt IgM motif from Walther et al. (28), and may include the reverse primer with an ultralong CDR3 (defined as up to 220 nt between the pre- and post-CDR3 motifs)
2	Forward primer	AGATGAACCCACTGTGGACC	Four or fewer mismatches	Using SeqKit, search for the forward primer with four or fewer mismatches, which allows there to be up to 4 nt differences from the initial motif searched	Four mismatches were allowed while matching the forward primer. This allowed lenient filtering for maximum sequence capture for the first filtering steps. Sequences matching with four mismatches were in the correct location and resembled the forward primer
3a	IgM motif	ACAGCCTCTCT	Two or fewer mismatches	Using SeqKit, search for the IgM motif adapted from Walther et al. (28)	Two mismatches were allowed while matching a truncated version of the IgM motif from Walther et al. (28). This allowed lenient filtering to allow for maximum sequence capture. A truncated version was used because this motif is located farther downstream near the reverse primer, and sequences may potentially be shortened
3b	IgM motif	AATCACACCCGAGAGTCTTC	Four or fewer mismatches	Using SeqKit, search for the IgM motif from Saini et al. (4)	Four mismatches were allowed while matching the IgM motif from Saini et al. (4). This allowed lenient filtering for maximum sequence capture for the first filtering steps. Sequences matching with four mismatches were in the correct location and resembled the IgM motif
4	Reverse primer	TGTTTGGGGCTGAAGTCC	Four or fewere mismatches	Using SeqKit, search for the reverse primer and return in the forward orientation. The presence of the reverse primer motif confirms the sequence to be IgM in origin	Four mismatches were allowed while matching the reverse primer. This allowed lenient filtering for maximum sequence capture for the first filtering steps. Sequences matching with four mismatches were in the correct location and resembled the reverse primer
5a	Merge file	—	—	Merge files from steps 3a and 3b and remove duplicate sequences	Searching two separate IgM motifs and combining the files allowed retention of as many sequences as possible for CDR3 identification
5b	Merge file	—	—	Merge files from steps 4 and 5b and remove duplicate sequences	This step allowed the combination of files of sequences that were all in forward orientation
6a	Pre-CDR3	GA[AG]GA[TC][ATG][GC][ATGC]GC[GCTA][ATC][CGT][ATCG].*	Steps 6a and 6b were run simultaneously using the “or” regular expression	All variations of step 6 were searched using SeqKit with regular expressions (regex) Search the pre-CDR3, trimming the sequences to the matched search motif The international ImMunoGeneTics information system (IMGT) was used as the basis of nucleotide and amino acid numbering and consulted for acceptable pre- and post-CDR3 sequence (Lefranc [29])	See Supplemental Excel file for illustration of alternative nucleotides in filtering steps 6 and 7 Searches in steps 6a and 6b were designed after completing a sequence alignment of previously published sequences and published bovine V segments from IMGT. Further optimization was completed following manual review because there were sequences that were filtered out due to consistent single mismatches
6b	Pre-CDR3	[ATG]C[GA]GCC[AG][CT][AG][TC]A[CT].*		Search the pre-CDR3, trimming the sequences to the matched search expression	
7a	Post-CDR3	[ATG][GC][ATGC]GC.*?TGGGG[GC]C[GCA][AG]	Steps 7a and 7b were run using the “or” regular expression at the same time	All variations of step 7 were searched using Python and regular expressions This search term captures the trimmed sequences from step 6 at the same location upstream of the CDR3, so CDR3 length can be calculated from the same location on every sequence. The “.*?” captures anything that follows the first search term until the second motif is identified. The regular expression ensures it captures the closest match to the first motif (distance based)	Captures potential variation in *IGHJ1-2*, *IGHJ1-3*, *IGHJ1-6*, *IGHJ2-2*, *IGHJ2-3*, and *IGHJ2-4* and allows variation in the third nucleotide of the glycine codon. During initial optimization, when analyzing sequences that were not captured, a common occurrence was the addition of a G nucleotide following the W118 position
7b	Post-CDR3	[ATG][GC][ATGC]GC .*?TGGGCCAA		A post-CDR3 motif coding for 1 nt loss	This search term incorporates sequences that were very similar to the segments in step 7a; however, when analyzing sequences that did not match the filtering of the published J segment sequence at this location, a consistent feature was a loss of a G nucleotide at this location, which still allowed for tryptophan at the W118 position
7c	Post-CDR3	[ATG][GC][ATGC]GC .*?CCGGGCCAA		The post-CDR3 search term here codes for proline in the W118 location instead of the tryptophan	During optimization, it was initially found that sequences were not matching the post-CDR3 search because there was a proline at the W118 location. The functionality of a proline in this location has not been evaluated; however, this may be an artifact of sequencing
7d	Post-CDR3	[ATG][GC][ATGC]GC .*?TGCGGCCGA		This post-CDR3 search motif searches for the *IGHJ2-6* J segment	This search motif was derived from bovine J segments from IMGT
7e	Post-CDR3	[ATG][GC][ATGC]GC .*?TGGGGTC[AG]G		This post-CDR3 search motif searches for the *IGHJ1-5* and *IGHJ2-5* J segments	This search motif was derived from bovine J segments from IMGT
7f	Post-CDR3	[ATG][GC][ATGC]GC .*?TGTGGCCAG		This post-CDR3 search motif searches for the *IGHJ2-1* and IGHJ1-1*03 J segments	This search motif was derived from bovine J segments from IMGT and allowing for an allelic form of *IGHJ1-1*
7g	Post-CDR3	[ATG][GC][ATGC]GC .*?TGGGGCTCA		This post-CDR3 search motif searches for the IGHJ1-4*02 J segment	This search motif was derived from bovine J segments from IMGT and allowing for an allelic form of *IGHJ1-4*
7h	Post-CDR3	[ATG][GC][ATGC]GC .*?AGGGGCCAA		In this search term, the AGG would code for an arginine instead of the typical tryptophan seen at the W118 location.	This motif was included because it was found that sequences that did not match the filtering of the published J segment sequences commonly had AGG instead of TGG (tryptophan). Although the functionality of an arginine at this location is unknown, it allows for single nucleotide mismatches that may be an artifact of sequencing
7i	Post-CDR3	[ATG][GC][ATGC]GC .*?TGGAGCCAG		This post-CDR3 search motif searches for the IGHJ1-1*01 and IGHJ1-1*02 J segment	This search motif was derived from bovine J segments from IMGT and allowing for an allelic form of *IGHJ1-1*
8	tsv file	—	—	Generate a tsv file of DNA sequences with matched pre- and post-CDR3 motifs Twenty-seven nucleotides are subtracted from the length of CDR3	The generation of the tsv document provides the length of the matched CDR3 nucleotide sequence Twenty-seven nucleotides were subtracted so that the length of CDR3 was adjusted for the pre- and post-CDR3, so they are excluded from the length estimation
9	Translate	—	—	Translate matched CDR3 sequences to amino acid codons using SeqKit	
10	Remove stop codons	—	—	Remove sequences with stop codons from the analysis	
11	tsv file	—	—	Generate a tsv file with amino acid sequence of productive CDR3s Nine amino acids are subtracted from the total length	The generation of the tsv files provides the length of the productive CDR3 amino acid length to adjust for the pre- and post-CDR3 length, so C104 and W118 are not included in the length estimate

Each step includes the search step, filtering motif if applicable, the parameters used, the description of the step, and the motivation for the step/parameter.

#### Filtering

Filtering the sequence motifs of interest was completed using SeqKit (v2.3.1) and will be referred to as the refined stepwise literature-based (RSLB) filtering method. The first filtering step was for the forward primer (20 nt, 5′-AGATGAACCCACTGTGGACC-3′) with the option of up to four mismatches (Table I). The second step was searching for the reverse primer (5′-TGTTTGGGGCTGAAGTCC-3′) with the option for four mismatches. All matched sequences were returned in the forward orientation. After searching for the forward and reverse primers, the IgM isotype motifs (20-nt IgM motif) from Saini et al. (4) (5′-AATCACACCCGAGAGTCTTC-3′) with four mismatches and a truncated version of the 21-nt IgM motif from Walther et al. (28) (5′-ACAGCCTCTCT-3′) (with the option of two mismatches) were searched. The option for two or four mismatches was optimized and refined for each filtering search motif based on the number of sequences and the nucleotide sequence that was included or excluded at each step, which maximized capture of relevant sequences. Finally, files were merged, and duplicate reads were removed to create a file with forward orientation sequences that included both a forward primer and IgM motif.

The pre-CDR3 was defined as the FWR3 sequence immediately upstream from the CDR3, similar to the following sequence: 5′-GAGGACACGGCCACATACTACTG-3′. A search for the pre-CDR3 was completed using notarized bovine V genes obtained from the international ImMunoGeneTics information system (IMGT; https://www.imgt.org/HighV-QUEST) (Table I). The pre-CDR3 motifs were combined into two search motifs that would maximize capture of *IGHV* genes in the FWR3. After identifying sequences with a pre-CDR3, a search was carried out for post-CDR3 motifs immediately downstream of the CDR3 in FWR4, similar to 5′-TGGGGCCAA-3′. The search motif was designed using notarized J genes from the IMGT in the FWR4 and common nucleotide sequences to this area (Table I). Both pre- and post-CDR3 search motifs were adjusted to allow nucleotide substitutions on the basis that they were commonly present in sequences that did not match the initial CDR3 search. Regular expressions (regex) were used in SeqKit to identify sequences with specific variations of nucleotide patterns in the pre- and post-CDR3 (Supplemental Fig. 2). A tsv file was generated of DNA sequences that matched the pre- and post-CDR3 search, and the file included sequence match, length of matched sequence, and the sequence header. A Nextflow (v23.04.3) pipeline was implemented to complete all steps from FASTQC to the generation of the tsv file (step 10 in Table I) of filtered and matched DNA sequences. All scripts for the primary analysis and RSLB filtering can be found at https://github.com/harohodg/filtering_bovineIgM_rep.

#### Sequence analysis

After the CDR3s were identified, the amino acid length was calculated from C104 (in the V region) to W118 (beginning of the J region in FWR4), using the numbering system according to IMGT (29). C104 and W118 were not included in the calculation for CDR3 length. CDR3s that were ≤3 or ≥225 nt were removed because these lengths would not correspond to a CDR3 of 1–75 aa, which was established as an arbitrary cutoff from previous literature. Sequences longer than 75 aa typically matched much farther downstream (within the C region) where the typical post-CDR3 is located. The total numbers of sequences and ultralong CDR3s (≥40 aa) were counted. The total number of sequences with an identified CDR3 was used as the denominator to estimate the percentage of ultralong CDR3s.

**FIGURE 1.**
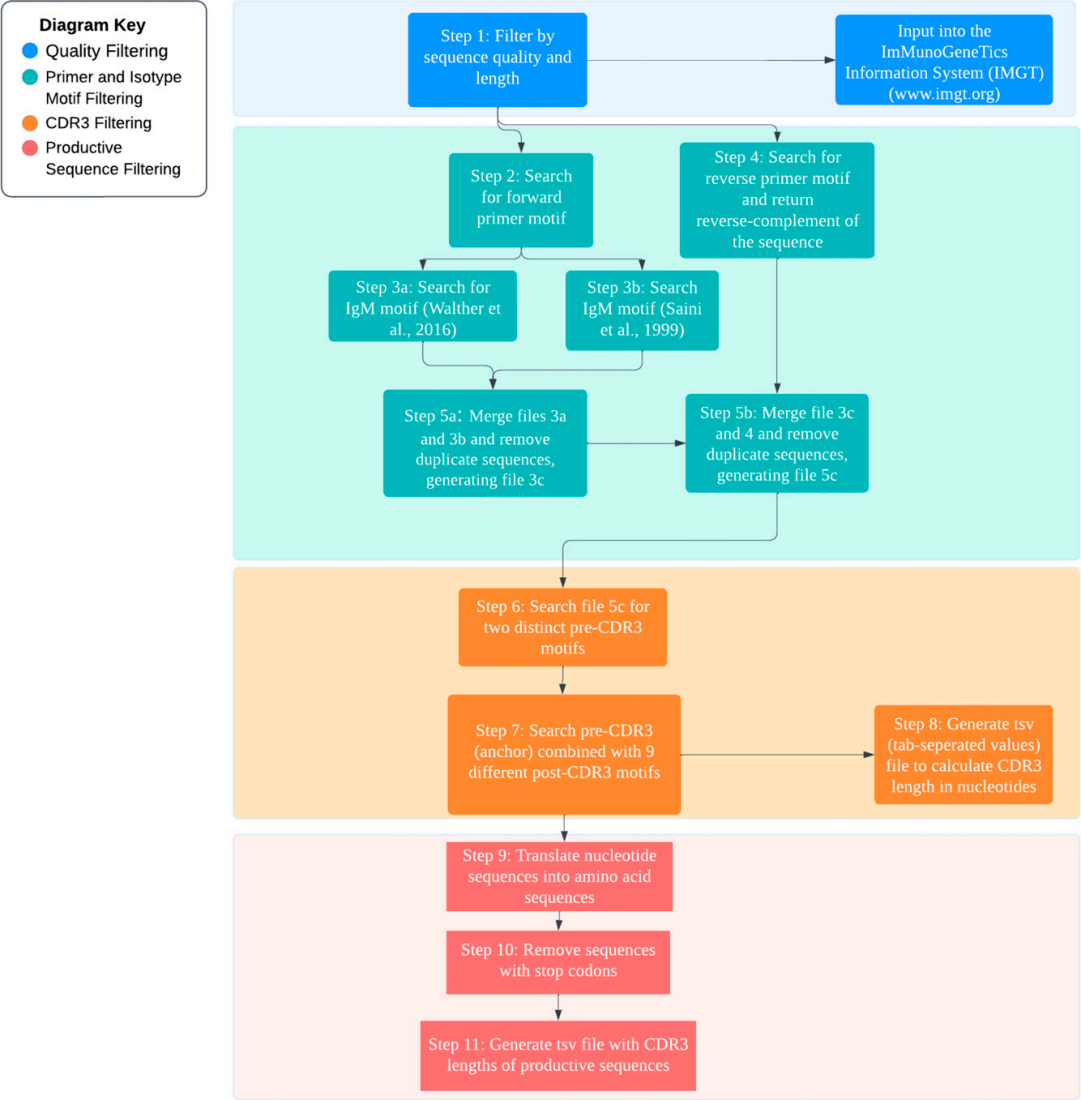
Flowchart of the refined stepwise literature-based filtering steps involved in the Nextflow pipeline. The pipeline begins with quality filtering, followed by primer and isotype motif filtering, and then CDR3 filtering. The sequences are then translated to protein sequences, and stop codons are removed. For a detailed description of each step, see Table I.

Productive sequences were also analyzed. First, using the filtering steps described above to isolate the CDR3, the sequences were translated using SeqKit from amino acid position 99 in FWR3 (upstream from CDR3) to 120 in FWR4 (downstream from CDR3). Sequences with stop codons were removed, and a tsv file was generated. Sequences with ≥40 aa from C104 to W118 were considered ultralong CDR3s. Sequences with ≤10 aa were considered short CDR3s. A summary flowchart of the RSLB filtering methods can be found in Fig. 1.

The distribution of CDR3 lengths was graphed using the ggplot2 function in R Studio. The IMGT HighV-QUEST (v3.6.0) software was used to validate the filtering and ultralong CDR3 estimations (30). IMGT HighV-QUEST provided VDJ labeling, gene usage, and CDR3 length estimation for total sequences and per clonotype (30). To assess the variation in the percentage of short, medium, and ultralong CDR3 length among cows, a multilevel χ^2^ test was used, and significant differences were reported at *p *<* *0.05. The Proc Corr function in SAS was used to compare the estimates of ultralong CDR3 percentages from RSLB and IMGT filtering and the percentage of ultralong CDR3s and *IGHV* gene usage.

To understand the reliability of ONT in measuring the length of the highly variable CDR3, the length of a 290-nt conserved IgM motif (GenBank accession no. AF005274.1) from the HC C region was measured and then the length of the conserved C region of IgM was measured in 77,499 MinION sequences. The conserved C region should remain the same length, which can provide an indication of the ability of ONT LRS to estimate the length of the CDR3. Sequences were matched using biopython with the following search term “TGAATCACACCCGAG.*?GGGTGATCGCTACA”. Once the length of the IgM sequence was measured, the distribution of IgM sequence length was graphed to visualize the difference in length of sequence to try to assess the commonality of artifacts.

## Results

**FIGURE 2.**
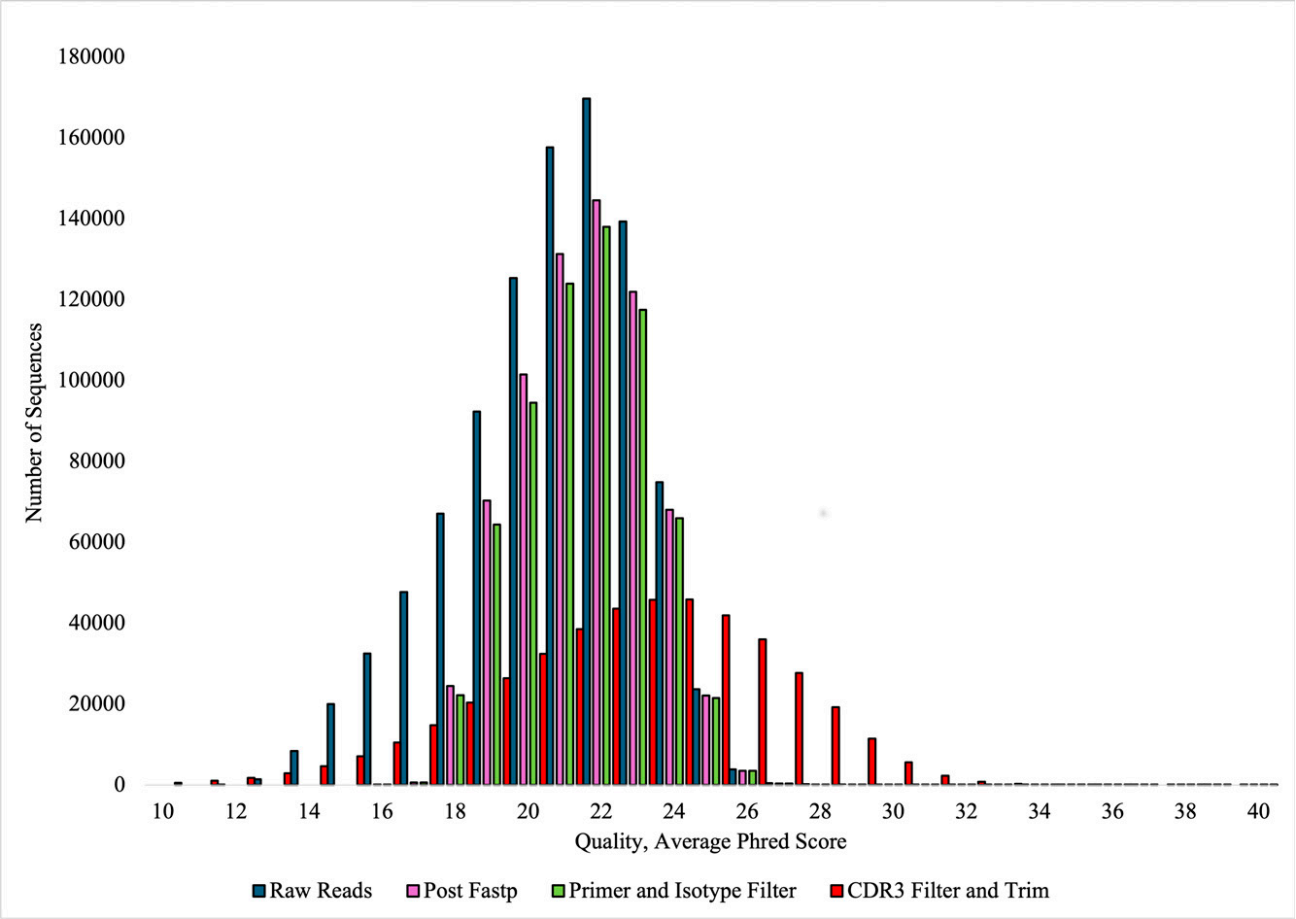
Average quality phred score per read and the number of sequences for each quality score for raw sequences, post–quality-filtered sequences, primer- and isotype-filtered sequences, and CDR3-filtered sequences. The quality score is calculated as the average phred score over the whole read. Only the CDR3-filtered and trimmed sequences received trimming of the length of sequence, which was completed to trim the sequence to the CDR3 motif.

**FIGURE 3.**
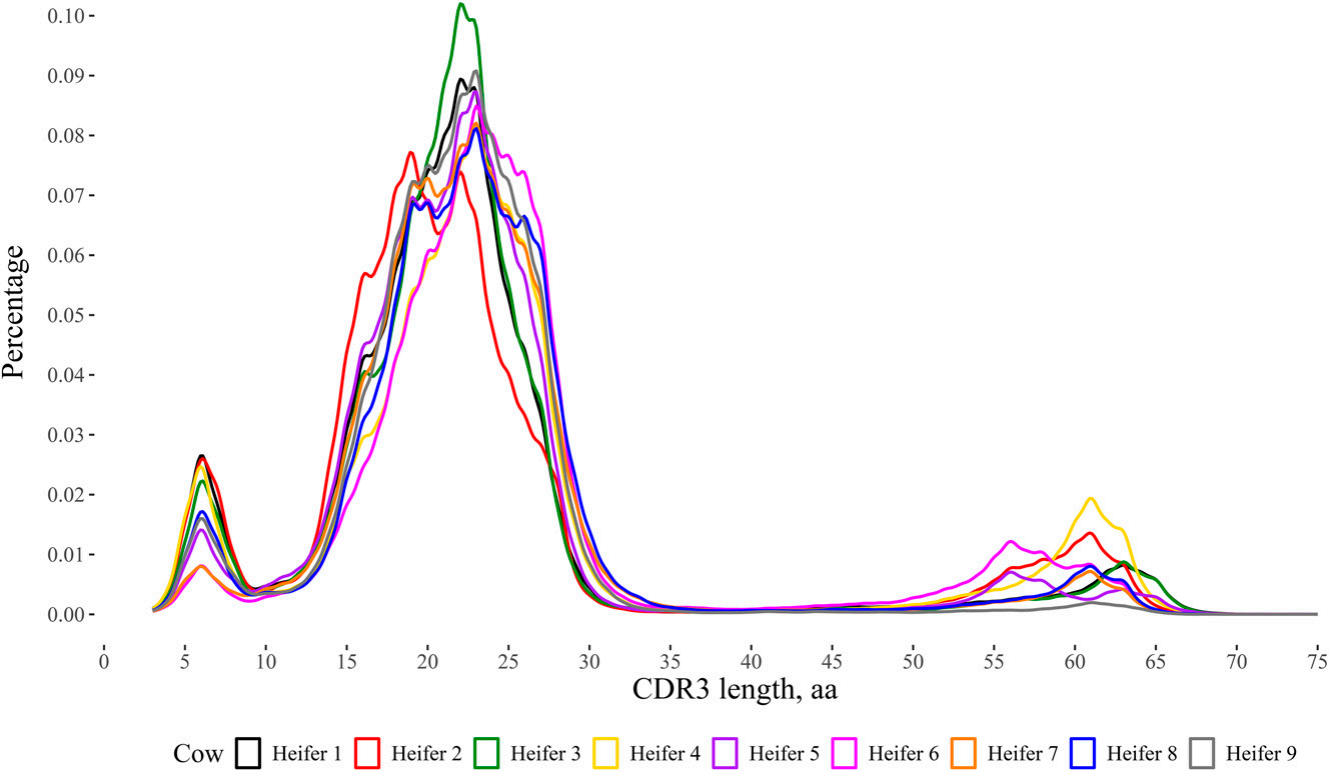
Distribution of CDR3 amino acid length in nine Holstein heifers. The kernel density plot was produced using the ggplot2 function in R studio, with a bandwidth of 0.5. Amplicons for IgM^+^ H chains were sequenced using the MinION R10.4 flow cell from Oxford Nanopore Technology. The refined stepwise literature-based (RSLB) filtering method was used to generate CDR3 lengths. In short, sequences were quality filtered and then sequences were filtered for the forward primer, reverse primer, IgM motif, and the pre- and post-CDR3. The CDR3 length was calculated for 261,386–444,031 sequences. The CDR3 was calculated from the framework region (FWR)3 starting after the cysteine at 104 to the amino acid before tryptophan at 118 found in FWR4 using the IMGT numbering scheme. Heifers varied significantly in the percentage of short, medium, and ultralong CDR3 lengths when analyzed with a multilevel χ^2^ test (*p* < 0.01).

**FIGURE 4.**
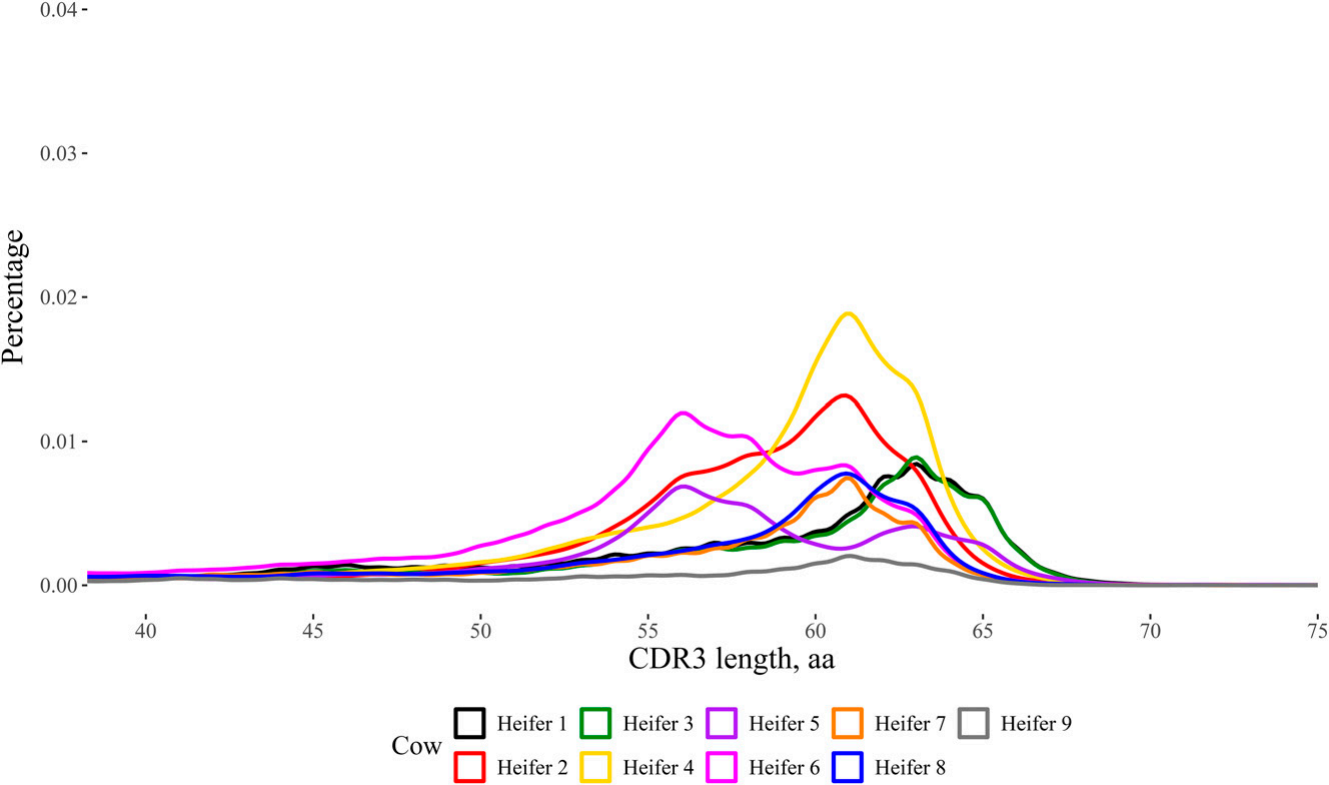
Distribution of CDR3 amino acid length in nine Holstein heifers in CDR3s with ≥40 aa. The kernel density plot was produced using the ggplot2 function in R studio, with a bandwidth of 0.5 and the *x*-axis beginning at 40 aa. Amplicons for IgM^+^ H chains were sequenced using the MinION R10.4 flow cell from Oxford Nanopore Technology. The refined stepwise literature-based (RSLB) filtering method was used to generate CDR3 lengths. In short, sequences were quality filtered and then sequences were filtered for the forward primer, reverse primer, IgM motif, and the pre- and post-CDR3. The CDR3 length was calculated for 261,386–444,031 sequences. The CDR3 was calculated from the framework region (FWR)3 starting after the cysteine at 104 to before the tryptophan at 118 found in FWR4 using the IMGT numbering scheme. Heifers varied significantly in the percentage of ultralong CDR3 length when analyzed with a multilevel χ^2^ test (*p* < 0.01).

The average number of raw sequence reads was 773,512 and ranged from 540,015 to 982,857 (Table II). For descriptive statistics on each step of filtering, see Table II. For graphics showing the change in average quality phred scores with filtering, see Fig. 2. Following filtering for length and quality, the number of sequences ranged from 392,107 to 697,581. After using the RSLB filtering method, which filters for the forward primer, reverse primer, IgM isotype motif, and pre-CDR3 and post-CDR3, the total number of sequences ranged from 261,497 to 444,363. Heifers varied significantly in the percentages of short, medium, and ultralong CDR3 length when analyzed via a multilevel χ^2^ test (*p* < 0.01, Figs. 3, 4). Ultralong CDR3s (≥40 aa in length) were present in 3.27–20.13% of IgM DNA sequences in individual heifers using the filtering techniques described above (Table III). The percentage of DNA sequences with short CDR3s ranged from 2.07 to 6.46% (Table III). The percentage of productive protein sequences with ultralong CDR3s was also investigated. Translating sequences derived from the RSLB filtering method and removing sequences with stop codons, the percentage of sequences with ultralong CDR3s ranged from 1.86 to 12.70% (Table III). The filtering method also provided short CDR3 (≤10 aa) estimates, ranging from 2.85 to 8.43% of sequences (Table III).

**Table II.** Descriptive statistics of reads before and after filtering

	Filtering	No. of Reads	Average Quality Score	Average Length	Length Distribution
Quality filtering	Raw data	965,022	20.73	1,131.9*^a^*	99–31,258
	Post fastp	688,856	21.56	998.5*^a^*	700–1,200
	Failed fastp	276,128	18.68	1,464.8*^a^*	99–31,258
Primer and isotype filtering	Forward primer	347,105	21.84	995.9*^a^*	700–1,200
	Failed forward primer	341,765	21.26	1,001.2*^a^*	700–1,200
	Reverse primer	311,140	21.30	1,003.3*^a^*	700–1,200
	Failed reverse primer	377,739	21.77	994.5*^a^*	700–1,200
	IgM motif (with forward primer)	343,326	22.01	996.9*^a^*	700–1,200
	Failed IgM motif	3,803	20.60	900.2*^a^*	700–1,200
	Combined	652,554	21.59	1,000*^a^*	700–1,200
CDR3 filtering and trimming	Pre-CDR3	557,455	21.65	625.9*^a^*	12–1,180
	Failed pre-CDR3	95,102	21.16	986.5*^a^*	700–1,200
	Pre- and post-CDR3	438,733 (Pre-length filter 441,750)	22.60	104.6*^a^*	1–225 (Pre-length filter 1–955)
	Failed pre- and post-CDR3	115,713	21.22	628.8*^a^*	12–1,164
Protein filtering and trimming	Productive protein sequence	318,878	—	31.6*^b^*	1–227

Descriptive statistics on the number of reads, average quality phred score, average sequence length, and length distribution of sequences at each step of filtering from heifer 1. These statistics include information on sequences that passed or failed a filtering step whether it was based on length, quality, or containing a search motif.

a Values for these sequences are reported on a nucleotide basis for average length and length distribution.

b Values for these sequences are reported on an amino acid basis for average length and length distribution.

**Table III.** Range of estimates of short CDR3s and ultralong CDR3s using RSLB filtering steps and IMGT resources with DNA sequences and productive protein sequences from nine Holstein heifers

	Filtering for Ultralong CDR3s (≥40 aa)	Filtering for Short CDR3s (≤10 aa)		IMGT Ultralong CDR3 Estimate (≥40 aa)	IMGT Short CDR3 Estimate (≤10 aa)
Total number of DNA sequences*^a^*	261,497–444,363			122,606–220,132	
Percentage of estimates from DNA sequences*^a^*	3.27–20.13 (12.62 ± 5.39)		2.07–6.46 (4.50 ± 1.60)	2.32–16.12 (9.73 ± 4.36)	2.24–8.22 (5.15 ± 1.86)
Total number of productive protein sequences*^b^*	190,993–318,603			121,594–201,578	
Percentage of estimates from productive protein sequences*^b^*	1.86–12.70 (7.60 ± 3.29)		2.85–8.43 (5.91 ± 1.96)	1.56–17.02 (7.15 ± 4.44)	2.93–8.84 (6.05 ± 2.16)

a Estimates of IgM H chain sequences with ultralong CDR3s and short CDR3s produced using DNA sequences from the refined stepwise literature-based (RSLB) filtering method described in *Materials and Methods* to search for sequences with the forward primer, reverse primer, IgM motif, and pre- and post-CDR3 motifs. In addition, ultralong CDR3s and short CDR3s were also estimated using the international ImMunoGeneTics information system (IMGT) from a DNA level. For the percentage estimates, the means and SD among the nine heifers are provided within.

b Estimates were produced using productive protein sequences. Productive sequences were determined with the refined stepwise literature-based (RSLB) filtering method described in the *Materials and Methods* and then translating sequences from the pre-CDR3 to post-CDR3 and removing sequences with stop codons. Productive sequences were also determined using the whole transcript identified by the IMGT, and then CDR3 length was calculated for productive sequences. For the percentage estimates, the means and SD among the 9 heifers are provided within the parentheses.

The established IMGT HighV-QUEST database–driven classification program was used to infer VDJ gene usage and estimate CDR3 length. Using IMGT, the percentage of ultralong CDR3s in sequences with one or more copy ranged from 2.32 to 15.55% and 2.37% to 16.12% for specific clonotypes (Table III). The percentage of short CDR3s using IMGT estimates ranged from 2.24 to 8.22%. Productive sequences were also investigated using full VDJ transcripts identified from IMGT (Table III). The percentage of productive ultralong CDR3s using IMGT ranged from 1.56 to 17.02%, and sequences with short CDR3s ranged from 2.93 to 8.84% (Table III). Based on the estimates using DNA sequences, the RSLB filtering method had a higher estimate of ultralong CDR3s, with the difference ranging from 0.95 to 4.58 percentage points. However, the estimates were positively and significantly correlated with IMGT estimates (*r* = 0.99, *p* < 0.01). Using the productive protein sequences, the RSLB filtering method has a higher percentage of ultralong CDR3s from 0.3 to 1.9 percentage points, except for the highest estimate, which IMGT estimated higher by 4.32 percentage points.

**FIGURE 5.**
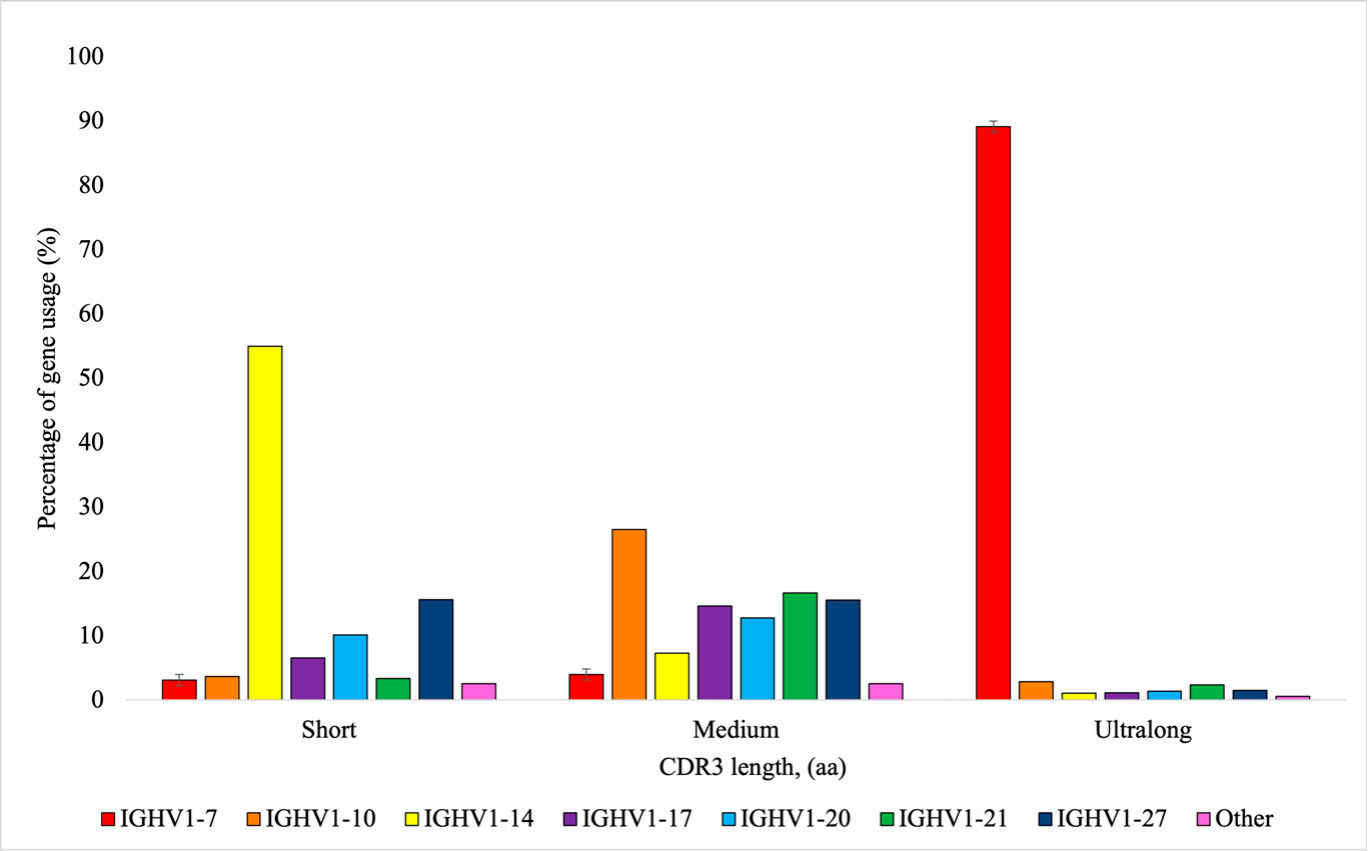
Percentage of the average V gene usage depending on the CDR3 length from nine Holstein heifers. Short CDR3s were considered ≤10 aa, medium CDR3s were >10 to <40 aa, and ultralong CDR3s were considered ≥40 aa. Amplicons for IgM^+^ H chains were sequenced using the MinION R10.4 flow cell from Oxford Nanopore Technology. VDJ labeling was completed using IMGT HighV-QUEST program.

**FIGURE 6.**
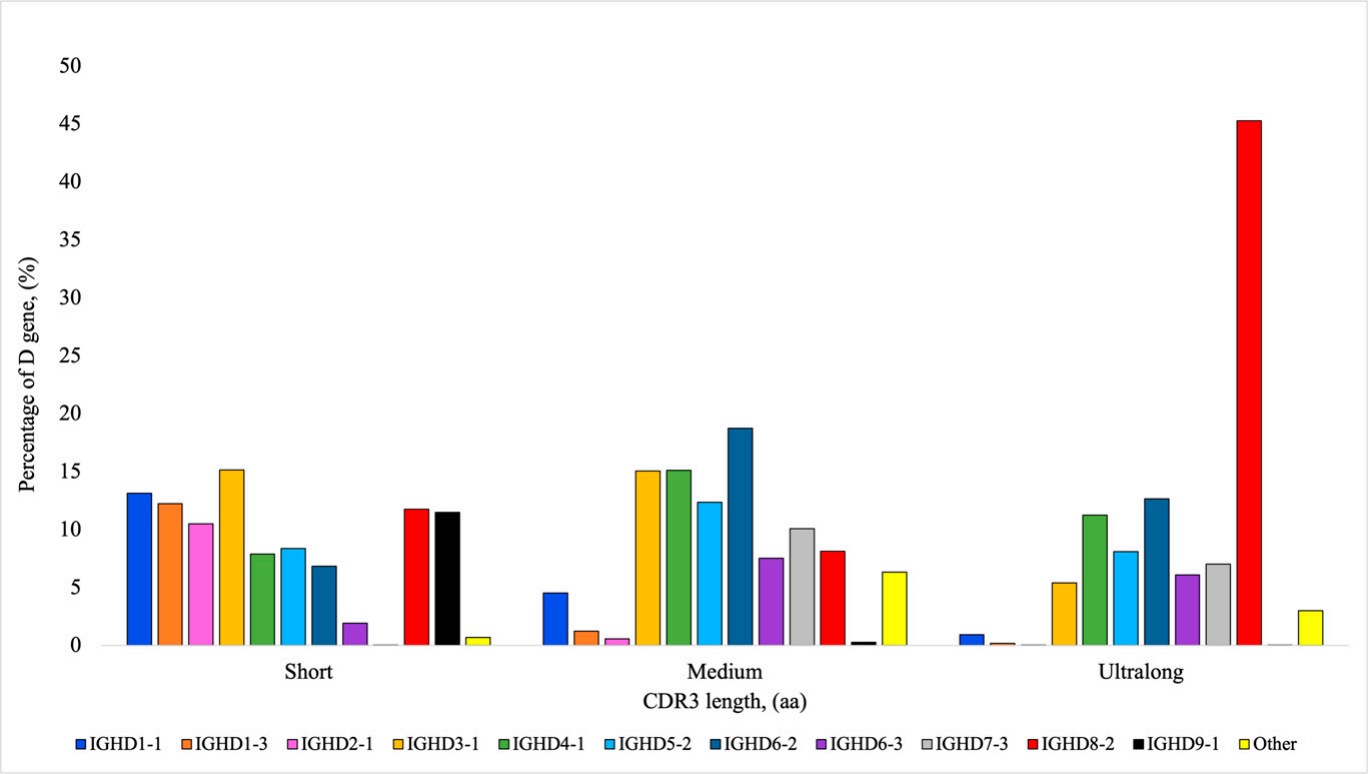
Percentage of average D gene usage depending on the CDR3 length from nine Holstein heifers. Short CDR3s were considered ≤10 aa, medium CDR3s were >10 to <40 aa, and ultralong CDR3s were considered ≥40 aa. Amplicons for IgM^+^ H chains were sequenced using the MinION R10.4 flow cell from Oxford Nanopore Technology. VDJ labeling was completed using the IMGT HighV-QUEST program.

**FIGURE 7.**
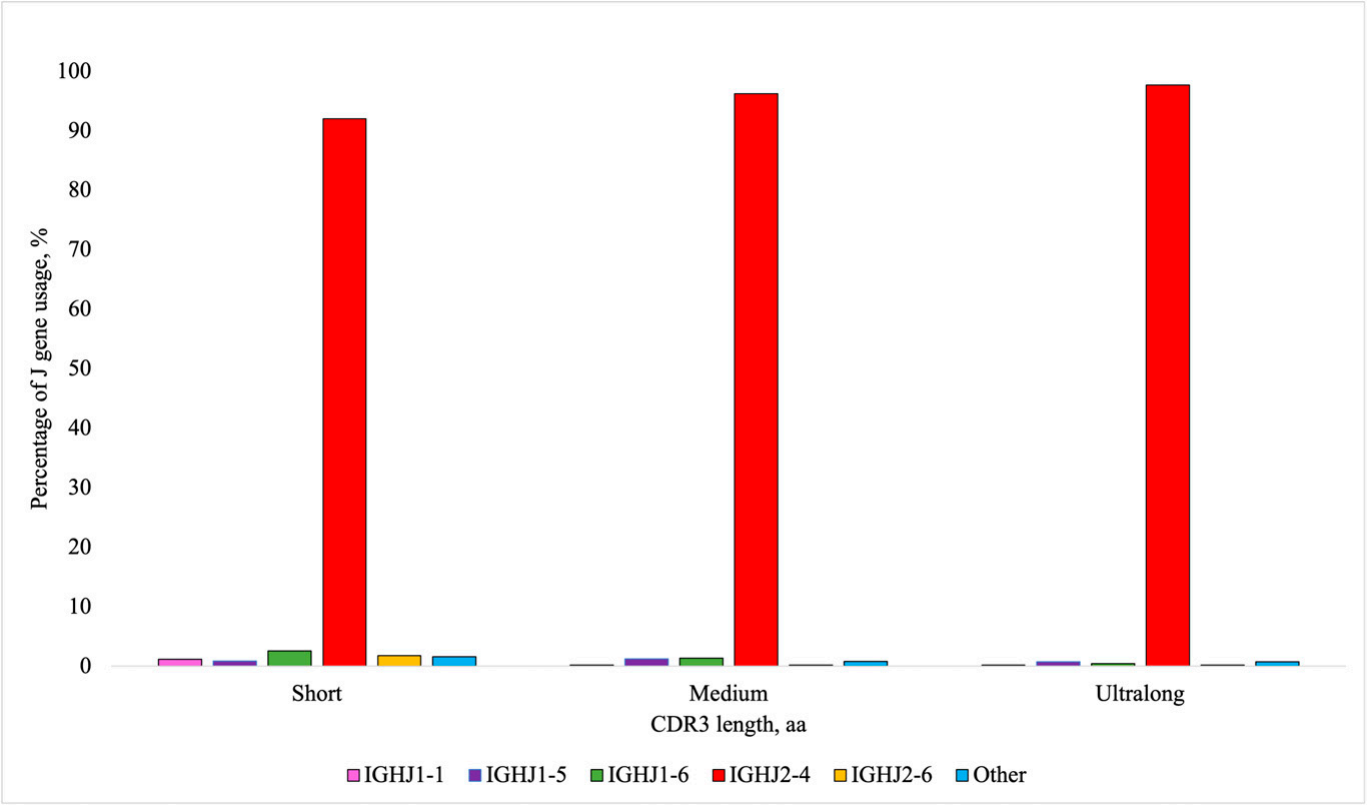
Percentage of average J gene usage depending on the CDR3 length from nine Holstein heifers. Short CDR3s were considered ≤10 aa, medium CDR3s were >10 <40 aa, and ultralong CDR3s were considered ≥40 aa. Amplicons for IgM^+^ H chains were sequenced using the MinION R10.4 flow cell from Oxford Nanopore Technology. VDJ labeling was completed using IMGT HighV-QUEST program.

According to the IMGT HighV-QUEST analysis, the V genes used most frequently in descending order included *IGHV1-10*, *IGHV1-7*, *IGHV1-21*, *IGHV1-27*, *IGHV1-20*, *IGHV1-17*, *IGHV1-14*, and *IGHV1-30*. The gene segment *IGHV1-7* was of interest due to its extensive use in ultralong CDR3 production (4, 12). Among heifers, the usage of *IGHV1-7* ranged from 5.8 to 24.2%. The usage of *IGHV1-7* and the percentage of ultralong CDR3s using the RSLB filtering method were positively and significantly correlated (*r* = 0.99, *p* < 0.01). The gene *IGHV1-7* was used in 89.11% (±0.88 SEM) of ultralong CDR3 sequences, with no other V segment being used more than 2.84% (Fig. 5). The usage of D genes varied by individual, with high usage of the following genes in descending order (>10%): *IGHD6-2*, *IGHD4-1*, *IGHD3-1*, *IGHD8-2*, *IGHD5-2*, and *IGHD7-3*. Usage of *IGHD8-2*, which is reported to be preferentially used for ultralong CDR3s, ranged from 9.65 to 15.55% in heifers. Notably, *IGHD8-2* was used in 45.29% (±1.50 SEM) of ultralong CDR3 sequences (Fig. 6). *IGHD6-2* and *IGHD1-4* were used in 12.67% (±0.39 SEM) and 11.24% (±0.28 SEM), respectively, with no other genes being used more than 8.11%. In contrast to V and D gene usage, J gene usage was consistent among animals. There was high usage of the *IGHJ2-4* gene, which varied among heifers from 95.1 to 96.3%. All other J gene usage was less than 1%, except for *IGHJ1-5* (0.83–1.02%) and *IGHJ1-6* (0.94–2.14%). *IGHJ2-4* was used in 97.66% (±0.15 SEM) of ultralong CDR3 sequences (Fig. 7). For short CDR3s, the *IGHV1-14* was highly used (54.96 ± 2.97%), followed by *IGHV1-27* (15.63 ± 2.01%) and *IGHV1-20* (10.14 ± 1.02%). The D gene usage among short CDR3s was similarly shared by *IGHD3-1*, *IGHD1-1*, *IGHD1-3*, *IGHD8-2*, *IGHD9-1*, and *IGHD2-1*. The gene *IGHJ2-4* was used in 92.01% (±0.30 SEM) of short CDR3 sequences.

One concern about ONT is the reliability and ability to measure the highly variable CDR3 length accurately. To assess this, the length of a 290-nt conserved IgM motif (accession no. AF005274.1) from the HC C region was calculated from 77,499 sequences. The distribution of the constant IgM region was then graphed and can be found in Supplemental Fig. 3. The mean length of the IgM C region of the matched sequences from this study was 287 nt long.

## Discussion

The findings in this study resulted in 261,497–444,363 sequences for analysis of the CDR3, which revealed a trimodal distribution of CDR3 length in all Holstein heifers. There was considerable variation among animals regarding the production of ultralong CDR3s, with ultralong CDR3s comprising 2.32–20.13% of DNA sequences and 1.56–17.02% of productive protein sequences, depending on the calculation method. Previously published estimates for the percentage of ultralong CDR3s are highly variable and range from 0.036 to 13% (4, 15, 16). This variation may be due to differences in cattle breeds, sample types, sequencing platforms, data analysis, and animal age. In addition, many of these studies were completed on a limited number of animals and with lower coverage of the BCR repertoire.

In this study, there was a subset of BCRs with short CDR3s (≤10 aa), which have received little attention in the literature. In cattle, it is not known if there is a difference in the functionality of the short CDR3s, but in humans and mice, short CDR3s have been associated with certain attributes. In humans, VRC01-class broadly neutralizing Abs typically have a short LC CDR3 (<5 aa), which is necessary to allow proper interaction with gp120 from HIV, producing a flexible Ag binding groove (25, 31). In addition, Havenar-Daughton et al. (25) reported a negative correlation between HC CDR3 length and naive VRC01 B cell affinity to a HIV candidate immunogen, used to target VRC01-class germline Abs. Generally, Rosner et al. (24) reported that Abs with short HC-CDR3s tend to use HC-CDR1 and HC-CDR2 to bind Ags.

Oyola et al. (7) reported a subset of short HC-CDR3s in Ankole and Boran cattle breeds. There was common usage of *IGHV1-14* in both breeds for short HC-CDR3s and usage of *IGHV1-20* and *IGHV1-27* in Ankole cattle for the production of short to medium HC-CDR3s (7). The current study found similar results, with high usage of *IGHV1-14*, followed by *IGHV1-27* and *IGHV1-20*. However, Deiss et al. (6) reported high usage of *IGHV1-10* for short and medium HC-CDR3s in Holsteins. Oyola et al. (7) reported that sequences with short- and medium-length HC-CDR3s had higher total number of mutations within CDR1, CDR2, and CDR3 compared with sequences with ultralong CDR3s. In sequences with ultralong CDR3s, mutations were limited to the CDR3 (7). For these reasons, the subset of bovine BCRs with short HC-CDR3s deserves further investigation to see whether there are functional differences for specific Ag interactions and differential VDJ gene usage.

In the current study, there was high usage of eight of the functional V genes *IGHV1-30*, *IGHV1-27*, *IGHV1-21*, *IGHV1-20*, *IGHV1-17*, *IGHV1-14*, *IGHV1-10*, and *IGHV1-7*. A study by Safonova et al. (32) focused on the same eight V genes due to their high usage. Previous literature has discussed the use of *IGHV1-7* for the production of ultralong CDR3s (4, 12), which was seen to be significantly associated with ultralong CDR3 production in this study and estimates of *IGHV1-7* usage for ultralong CDR3 resembled findings from the cited literature. Usage of different V genes likely differs among populations, breeds, and ages of animals. High usage of the *IGHJ2-4* (95.1–96.3%) was seen in all heifers, consistent with previous J gene usage findings (7). The reason for preferential usage of the J gene is unclear; however, a limited number of functional J genes are available in cattle. In the study by Oyola et al. (7), the researchers only found one other J gene with usage above 1%, which was *IGHJ1-6*. In the current study, both *IGHJ1-5* and *IGHJ1-6* had greater than 1% usage, but the highest usage of these genes was 2.14%.

There are limitations and potential errors in labeling VDJ gene usage. Labeling the V gene segments can be difficult since there is only one functional V gene family in cattle, which shares 90% sequence identity (6). There are also limitations in labeling D genes. The D gene provides most of the sequence within the CDR3, which is the highly variable area of the sequence. If the germline sequence has undergone SHM or has insertions in the junctions, it can reduce the ability to properly label D segments and increase the chances of labeling the gene incorrectly. The *IGHD8-2* gene also contains many repetitive nucleotide sequences, which can complicate gene labeling further.

To our knowledge, this is the first study to use ONT to sequence the bovine IgM repertoire. Previously, other technologies such as PacBio, Illumina, and Sanger sequencing have been used to analyze the BCR repertoire in cattle (4, 7, 32). The MinION used in this study provided good coverage depending on the animal. Oyola et al. (7) used a nested PCR strategy with an Illumina SRS platform. Using this strategy, they assembled 567,051 and 807,600 sequences from FWR1 to FWR4, starting with ∼2 million sequences (7). The most extensive study on ultralong CDR3s used a SRS platform to sequence samples from 204 pure-bred Angus calves at four time points; however, coverage information was unavailable (32). Many previous studies have used sequencing platforms such as Sanger sequencing (4, 28), which required intensive laboratory work and resulted in low throughput. For example, Saini et al. (18) investigated ∼44 sequences using heterohybridoma cells, and Walther et al. (28) analyzed 509 sequences from four different breeds using this method. The LRS platform by PacBio has been used but is ultimately low-throughput, expensive, and typically more error-prone than SRS (33). In one study using PacBio, there was a total of 49,945 sequences from four calves (15). In the current study, ∼50% of sequences were recovered using the CDR3 RSLB filtering method, which is comparable to, if not more successful than, previous methods. Compared with previous results from the literature, using the ONT MinION provided cost-efficient coverage without the need to assemble the CDR3 sequence from shorter reads. ONT offers the R10.4 flow cell for ∼$900 USD, which can be used with barcoding kits suitable for 24 (∼$699 USD) to 96 samples. Because the primary interest of this study is the CDR3, which is found within 800- to 1200-nt amplicons, the choice to use LRS helped avoid issues in assembling and interpreting reads, which may have occurred with the SRS technology that was available during this study. In the future, there will likely be more options for high-throughput technology that will be cost-effective and accurate.

In the future, other technologies such as RAGE-seq (a droplet-based single-cell long read RNA platform combining ONT and Illumina) and rolling circle to concatemeric consensus (R2C2) sequencing with the addition of unique molecular identifiers in laboratory workflows could be valuable for sequencing the repertoire. Future analyses will use anchor clustering to analyze clonotypes and clonotype relationships (34). The publicly available Immcantation packages may be useful to identify mutations and insertions in the CDR3 (https://immcantation.readthedocs.io/en/stable/#).

One of the limitations of this study is the accuracy of ONT MinION sequencing. Earlier versions of ONT had a reputation for lower accuracy, but with the newer R10.4 technology, there is improved accuracy (26, 27). How ONT copes with highly variable sequences such as the CDR3 sequence has yet to be elucidated. For this reason, discerning mutations due to SHM from base calling errors can be difficult, but the newest accuracy estimates are reassuring. Efforts have been put forward by Shlemov et al. (35) through a program called the Diversity Analyzer, which predicts sites of SHM versus germline nucleotide variation, which may prove valuable in the current context. Older versions of ONT (R9) are also prone to indels, especially in low-complexity or homopolymer sequences (26). However, the R10 ONT versions were designed to improve read quality over low-complexity areas. In this study, the accuracy of ONT’s ability to determine the length of the conserved C region of IgM was estimated by comparing the length of the C region with the published sequence length of the C region of IgM, and the distribution proved to be very similar.

Previously, the Nanopore R10.4 technology has been benchmarked against Illumina sequencing (26). A study by Byrne et al. (36) used ONT long-read RNA sequencing to complete a transcriptomic study on B cells in mice and compared their results with Illumina sequencing. Byrne et al. (36) found a significant and positive correlation between gene expression using Nanopore LRS and Illumina SRS. However, it has yet to be benchmarked for BCR repertoire data in cattle, which, in the future, would be valuable to validate and compare and contrast the findings.

Many studies of the bovine BCR repertoire have used different sequencing platforms with different sample types, primers, laboratory preparations, and populations. Ultimately, benchmarking against other platforms at this time may prove less valuable. For example, SRS can already introduce bias against ultralong CDR3s due to the nature of the sequencing platform (7). Oyola et al. (7) reported that there is a bias against ultralong CDR3 reads using SRS. Had they not implemented the nested PCR strategy, they would have underestimated the presence of ultralong CDR3 sequences and only recovered 4 and 2.6% of the CDR3s longer than 22 aa in Ankole and Boran breeds (7). Abs with ultralong CDR3s are unique to the *Bos* and *Bison* genera, and because of their length, historic sequencing methods have limited and biased the sequencing and identification of ultralong CDR3s. Therefore, the LRS provides much more than just another method to identify sequences with ultralong CDR3s but will allow a more accurate representation of the repertoire and allow identification of more unique sequences that would otherwise be missed by previous methods.

There is also a potential bias inherent in transforming sequences to productive protein sequences. In the current study, there was a loss of 22.6–31.3% of sequences when translating sequences to productive protein sequences. Ultimately, the percentage of ultralong CDR3s likely decreased once sequences were translated to productive protein due to the increased length of the CDR3, which increases the probability of a random nucleotide error resulting in a stop codon. However, other studies had similar losses in the number of sequences. Larsen et al. (15) lost 26.8% of sequences when translating to productive protein sequences. Oyola et al. (7) lost 32 and 14% of sequences when translating to productive protein sequences from DNA sequences in Ankole and Boran breeds.

Another limitation of this study is that the sample size was relatively low; only nine animals were used. However, previous studies typically used only one to four animals (4, 7, 15). Only one study has used more than four animals with good coverage of the Ab repertoire, which used 204 purebred black Angus calves using a SRS platform (32). Few studies have examined breed differences in the prevalence of ultralong CDR3s (7, 28). The current study was conducted on a closed purebred Holstein research herd from heifers in a limited age range. The results may be specific to this population and may not represent other breeds, herds, and ages. Another limitation was that this study used PCR to amplify sequences of interest to ensure appropriate coverage of the IgM repertoire. However, the same PCR bias was applied to all samples. It may be essential in future analyses to continue including the IMGT clonotype estimates to reduce potential PCR bias. Unique molecule identifiers can also be incorporated into the laboratory workflow to help distinguish which sequences are derived from a clonotype or which constitute copies of the same amplicon from PCR. Sorting of IgM^+^ cells was carried out to enrich the B cell population for RNA extraction and increase the yield and efficiency of PCR.

The depth of sequencing required to provide a good representation of the BCR repertoire is somewhat ambiguous. The B cell repertoire is a snapshot into the B cell response at a given point in time, and intersample variation is not currently understood for this dataset. The functional VDJ repertoire in cattle can allow for ∼768 unique HC recombination events. There are even further opportunities for diversification through P- and N-nucleotide additions at junctions and SHM events within CDRs. The ability to sample every potential B cell at a given time point is unattainable. In humans, individuals may have ∼3 × 10^11^ B cells, making it difficult to provide a complete representation of the repertoire (37).

The findings from this study give reason to continue to use the MinION for bovine BCR sequencing. Heifers within this study showed variation in the percentage of ultralong CDR3s, which warrants further study on factors contributing to this variation. Variation was previously seen in other studies among individual animals on a genetic basis and an influence of vaccination (32). Future studies plan to look at the BCR repertoire of cattle through life stages and life transitions. Understanding the influence of pathogen pressures and vaccination will be valuable during early life and major transitioning periods. Establishing how BCR genetics plays a role in disease resistance within individual animals will provide opportunities to include traits in breeding schemes for healthier animals with improved vaccine responses.In conclusion, the bovine Ab repertoire is remarkable due to the presence of ultralong CDR3s, which were found to compose 2.24–20.13% on a DNA basis and 1.56–17.02% as productive protein sequences of the Ab repertoire in this study. Due to the length of the CDR3, there are limitations to using SRS platforms, such as assembling fragments spanning the hypervariable region, which is why a LRS platform was chosen for this study. As there are continuous improvements in sequencing technology, the efficiency and cost of sequencing continue to reflect this. Oxford Nanopore’s MinION provided ample IgM^+^ sequences and, after filtering and analysis, provided results consistent with previous literature findings generated using platforms such as Illumina and PacBio. In the future, ONT R10.4 technology can be used to provide an efficient and cost-efficient method of sequencing PCR-amplified BCR repertoires and estimating the percentage of ultralong CDR3s.

## Supplementary Material

Supplemental Material 1 (PDF)

Supplemental Material 2 (XLSX)
