# Supplementary material for: Estimates of Sequences with Ultralong and Short CDR3s in the Bovine IgM B Cell Receptor Repertoire Using the Long-read Oxford Nanopore MinION Platform
Source: Immunohorizons. 2024 Sep 9;8(9):635–51. doi: 10.4049/immunohorizons.2400050 (PMC11447701; doi:10.4049/immunohorizons.2400050)
Supplement: Supplemental Material 1 (PDF) [file IH_2400050_Supplemental_1.pdf]

Supplementary File:

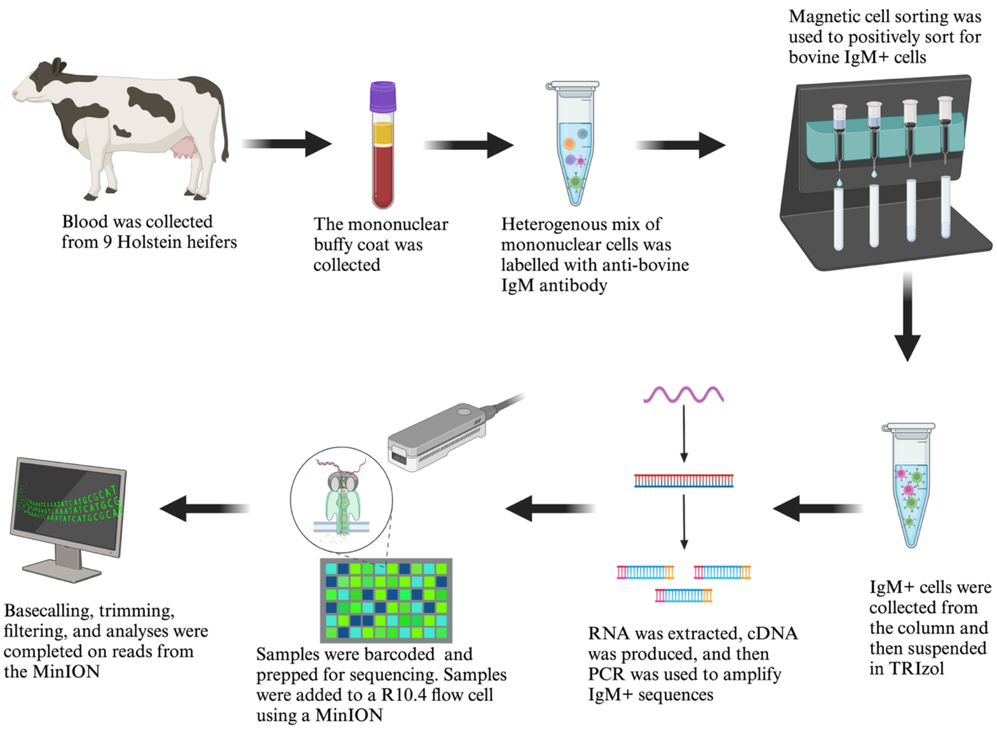

Supplementary Figure 1. Flow chart of laboratory methods produced in BioRender with a publishing licence

a) Search term using regular expressions:

```
G
A C G GCC A C A T A C .*?TGGGGCCAA
T   A     G T G C   T
```

[ ] - match any character within the brackets
. - any character
* - search 0 or more instances after the preceding regex motif
? - find the motif 0 or 1 times at most

b) Matches in the sequence

```
AGATGAACCCACTGTGGACCCTCCTCTTTGTGCTCTCAGCCCCCAGAGGGGTCCTGTCCAGGTGGCTGGAGGAAGTCGGGCCCCAGCCTGGTGAACCCTGCA
CAGACCCCTCTCCCTCGCCCTTGCACGGTCTCTGGATTCTGATTGAGCAACTATGCTGTAGGCTGGGGTCCAGCCAGGCTCCGGGGAAGGCGCTGGAGTCAT
TGGTGGTATGAGTATTAATGGAAAGACATGCCTTAACCCAGCCCTGAAATCCCGGCTCAGCATCACCAAGGACAACTCCAAGAGCCAAGTCTCCCTATCAGT
GAGCAGCGTGACACCTGAGGACACGGCCACATACTACTGTGCGAGGTGTTACTCCGGTGGTGAGACTAGTCCAACTTGTGGCGTTTCTGCTGATGGTGCGGG
GGTCGAAGATGTTGGGGCCAAGGACTCCTGGTCACCGTCTTCAGAAGGTGAATCACACCCGAGAGTCTTCCCCTGGTGTCCTGCGTGAGCTCGCCATCCGAT
GAGAGCACGGTGGCCCTGGGCTGCCTCGCCCGGGACTTCGTGCCCAAATTGAGTCAGCTTCTCCTGGAAGTTCAACAACAGCACAGTCAGCAGCGGAGGAAT
TTCTGGACCTTCCCCGAAGTCCTGAGGGACGGCTTGTGGTCGGCCTCCTCTCAGGTGGTCCTGCCCTCCTCAAGCGCCTTTCAAGGGCCGGATGACTACCTG
GTGTGCGAAGTCCAGCACCCCAAGGGAGGAAGAACCATGGCACCGTGAGGGTGATCGCTCAGAGTGAGTCAGAGGCGGAAGTGCTGTCCCCAGTCGTGAGTG
TCTTTGTCCCGCCTCGCAACAGCCTCTCTGGTGACGGCAATAGCAAGTCCAGCCTCATCTGCCAGGCCACGGACTTCAGCCCCAAACA
```

Supplementary Figure 2. a) The search term using regular expressions (regex) to define the pre-CDR3 and post-CDR3 regions. The first motif contains the pre-CDR3; within [] are options for search matches at that nt location. The nucleotides highlighted in bold red are the most common nucleotides for that location in the V gene. Brackets allow a ‘wild card’, meaning it can be any option within the brackets if it matches the rest of the search motif. The following term “.*” tells seqkit that this is a string and to search for the first motif in combination with the second motif only if the first motif precedes the second motif. Adding the “?” indicates a "non-greedy argument", telling seqkit to search for these motifs and take the first match found. b) Example result of a search result using regex. The matched pre-CDR3 in FWR3 and post-CDR3 found in FWR4 are highlighted in magenta. Nucleotides in the blue highlighted CDR3 region are counted. The forward and reverse primers are highlighted in yellow, the 21 nt IgM motif from Walther et al., 2016 (28), is highlighted in green, and the 20 nt IgM motif from Saini et al., 1999 (4) is highlighted in red.

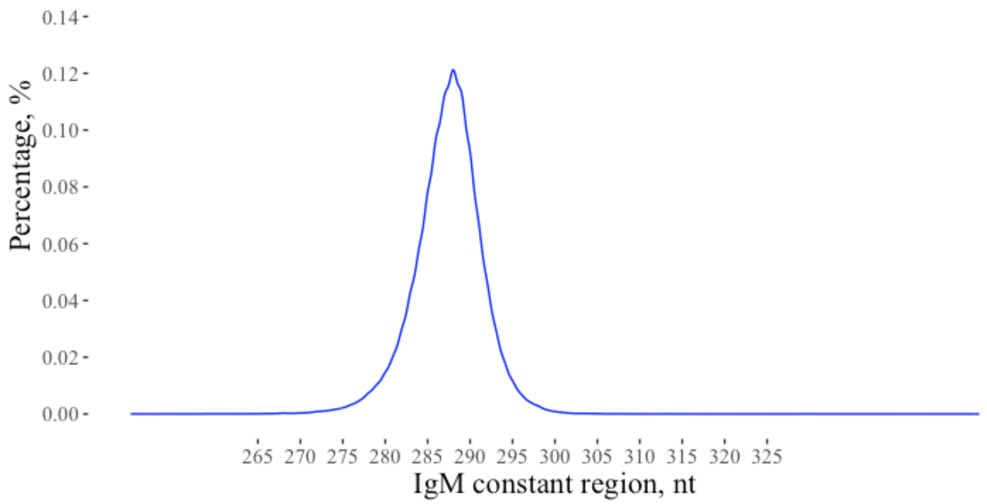

Supplementary Figure 3. The distribution of the length of the conserved constant region of IgM was measured in 77,499 MinION sequences. The conserved constant region should remain the same length, which can provide an indication of the ability of ONT LRS to estimate the length of the CDR3 compared to a conserved 290 nucleotide IgM motif (GenBank accession # AF005274.1). The mean length of the IgM constant region from the data sequenced using ONT was 287 nucleotides.
